# Validation of the acoustic change complex (ACC) prediction model to predict speech perception in noise in adult patients with hearing loss: a study protocol

**DOI:** 10.1186/s41512-024-00164-6

**Published:** 2024-01-23

**Authors:** Lana Biot, Laura Jacxsens, Emilie Cardon, Huib Versnel, Koenraad S. Rhebergen, Ralf A. Boerboom, Annick Gilles, Vincent Van Rompaey, Marc J. W. Lammers

**Affiliations:** 1 Resonant labs Antwerp, Department of Translational Neurosciences, Faculty of Medicine and Health Sciences, University of Antwerp, Antwerp, Belgium; 2 Department of Otorhinolaryngology, Head and Neck Surgery, Antwerp University Hospital (UZA), Edegem, Belgium; 3 Department of Otorhinolaryngology and Head & Neck Surgery, University Medical Center Utrecht, Utrecht University, Utrecht, the Netherlands; 4 UMC Utrecht Brain Center, Utrecht University, Utrecht, the Netherlands

**Keywords:** Hearing loss, Hearing impairment, Speech perception, Speech in noise, Biomarker, Acoustic change complex, Evoked potential, Objective measurement, EEG, Electroencephalography

## Abstract

**Background:**

Speech perception tests are essential to measure the functional use of hearing and to determine the effectiveness of hearing aids and implantable auditory devices. However, these language-based tests require active participation and are influenced by linguistic and neurocognitive skills limiting their use in patients with insufficient language proficiency, cognitive impairment, or in children. We recently developed a non-attentive and objective speech perception prediction model: the Acoustic Change Complex (ACC) prediction model. The ACC prediction model uses electroencephalography to measure alterations in cortical auditory activity caused by frequency changes. The aim is to validate this model in a large-scale external validation study in adult patients with varying degrees of sensorineural hearing loss (SNHL) to confirm the high predictive value of the ACC model and to assess its test–retest reliability.

**Methods:**

A total of 80 participants, aged 18–65 years, will be enrolled in the study. The categories of severity of hearing loss will be used as a blocking factor to establish an equal distribution of patients with various degrees of sensorineural hearing loss. During the first visit, pure tone audiometry, speech in noise tests, a phoneme discrimination test, and the first ACC measurement will be performed. During the second visit (after 1–4 weeks), the same ACC measurement will be performed to assess the test–retest reliability. The acoustic change stimuli for ACC measurements consist of a reference tone with a base frequency of 1000, 2000, or 4000 Hz with a duration of 3000 ms, gliding to a 300-ms target tone with a frequency that is 12% higher than the base frequency. The primary outcome measures are (1) the level of agreement between the predicted speech reception threshold (SRT) and the behavioral SRT, and (2) the level of agreement between the SRT calculated by the first ACC measurement and the SRT of the second ACC measurement. Level of agreement will be assessed with Bland–Altman plots.

**Discussion:**

Previous studies by our group have shown the high predictive value of the ACC model. The successful validation of this model as an effective and reliable biomarker of speech perception will directly benefit the general population, as it will increase the accuracy of hearing evaluations and improve access to adequate hearing rehabilitation.

## Background

Hearing impairment is the most frequent sensory deficit in humans, affecting almost 20% of the population worldwide. It has been listed by the World Health Organization as a priority disease for research into therapeutic interventions [1, 2]. Hearing loss not only affects communication and quality of life but also causes social distress and anxiety and has a negative impact on cognitive functioning [3–6]. Currently, no treatment is available to prevent or halt the progression of sensorineural hearing loss (SNHL). Management mainly consists of hearing rehabilitation with hearing aids and/or cochlear implants depending on the grade of hearing loss. Speech perception tests are essential to measure the functional use of hearing, to determine the effectiveness of hearing aid fittings, and to evaluate cochlear implant candidacy [7]. However, these language-based tests are influenced by linguistic and neurocognitive skills [8, 9]. In Belgium, and in particular in the Flemish region, validated speech perception tests are only available in the Dutch and French languages. For patients with insufficient proficiency in these languages, audiologists are unable to obtain a reliable evaluation of their functional hearing impairment. The lack of language-independent tests to assess auditory function and speech perception further restricts access to healthcare and adequate hearing revalidation with hearing aids or cochlear implants for adults as well as for children [10]. The consequences of untreated hearing loss in these vulnerable populations are far-reaching, worsening their social isolation and impeding their process of integration into society. The same holds for adults and children with intellectual disabilities in whom no conventional speech discrimination tests can be performed due to their limited cognitive abilities. In this population, genetic causes of hearing loss (syndromic and non-syndromic) are frequent, and adequate hearing rehabilitation is challenging due to their cognition, attention span, and cooperation. To overcome these challenges we want to introduce a new biomarker to predict speech perception in noise.

Speech perception is strongly associated with spectral shape discrimination [11, 12]. Unfortunately, frequency discrimination tests require active participation and can be challenging for those with hearing impairment. Therefore, we have developed a non-attentive model using the Acoustic Change Complex (ACC) to predict speech perception. The ACC is an auditory evoked potential revealing the cortical response evoked by changes within an ongoing stimulus [13].

For our prediction model, ACCs are evoked in response to 12% frequency increases (i.e., 1000–1120 Hz; 2000–2240 Hz; 4000–4480 Hz) at three base frequencies (1, 2, and 4 kHz) [14]. Multiple regression analysis for prediction of speech perception in noise (SRT; dB SNR) revealed that the strongest prediction model was obtained by averaging the obtained ACC latencies and average hearing loss measured with pure-tone audiometry at those three frequencies [14]. This model was able to explain 87% of the total variance, indicating that subjects with longer ACC latencies have worse speech perception in noise than subjects with comparable hearing thresholds and shorter ACC latencies. If HL was removed from this model, the combination of ACC amplitude and latency over those three frequencies still explained 74% of the total variance in speech perception in noise (*r*^2^ = 0.74, *p* < 0.001) [14]. The major advantage of the ACC over the currently available audiometric tests is that it is language-independent, and it does not require active participation of the listeners. Therefore, it can be used in patients with insufficient language proficiency and—potentially—cognitive impairment, or in cases where conventional audiometry is unreliable, or malingering is suspected. Moreover, the ACC would be most beneficial for evaluating the auditory abilities of the growing population of patients who struggle with healthcare accessibility due to language barriers.

In the current study, we aim to validate this ACC prediction model in a large-scale study. This project will be the first study to externally validate a highly promising prediction model for speech perception in noise. The results of this study are essential to confirm and validate the predictive potential of this new objective measurement, developed at UMC Utrecht [14] and is a prerequisite for clinical implementation.

## Methods

### Study design

**Fig. 1:**
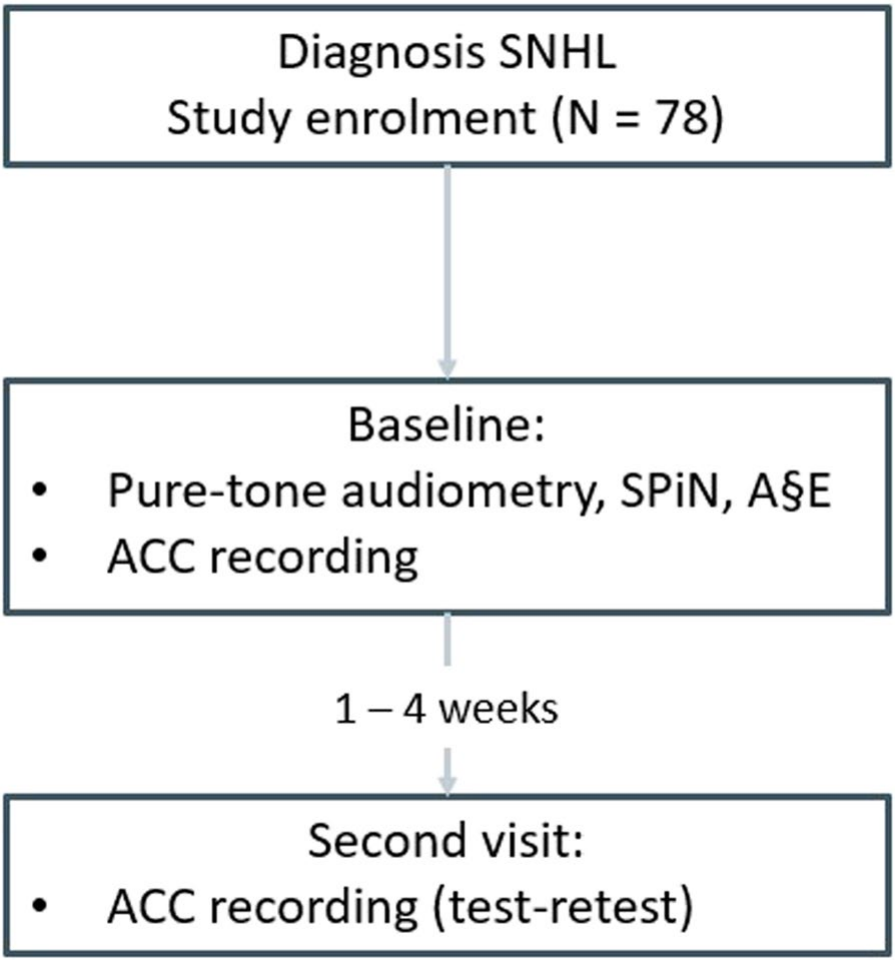
Protocol overview

Patients visiting the Antwerp University Hospital (UZA) Ear, Nose and Throat (ENT) outpatient clinic will be screened thoroughly for potential eligibility. If they meet the inclusion criteria and agree to participate, patients will be enrolled in the study (Fig. 1). The baseline evaluation will consist of pure-tone audiometry, speech-in-noise tests, a phoneme discrimination test, and a total of three ACC recordings. The entire test procedure will take about 2 h. Additionally, participants will be asked to fill in a questionnaire asking about their hand dominance, language proficiency, and musical experience. Recent literature has shown that professional musical training is beneficial for improving frequency discrimination and the ability to detect changes in frequency, leading to alterations in the ACC amplitudes [15, 16]. At the end of the first session, an appointment for the second session will be scheduled after 1 to 4 weeks.

During the second visit, the same three ACC recordings will be obtained for each patient. This allows us to assess the test–retest reliability of the ACC prediction model. Test–retest reliability is a valuable method for assessing the stability of the ACC prediction model over time. Pure tone audiometry, speech-in-noise testing, and a phoneme discrimination test will not be performed again during the second visit. In the brief timeframe spanning 1 to 4 weeks, significant alterations in pure-tone thresholds, speech-in-noise scores, or phoneme discrimination are not anticipated. If patients perceive a subjective change in hearing during the test interval, pure-tone audiometry will be repeated. In case of significant deviations in thresholds, patients will be excluded from the study.

To ensure that all audiometric tests and ACC recordings are performed in an identical manner for all participants, the investigators are trained prior to the start of the study and will adhere to a strict study protocol. All tests will be performed in the same order for all participants. One researcher will perform audiometric testing while another researcher will perform the ACC recordings. For each patient, the investigator performing the audiometric tests will be blinded from the ACC recordings and vice versa to prevent potential bias.

### Participants

Study participants will be consecutively sampled from patients visiting the Antwerp University Hospital (UZA) ENT outpatient clinic for evaluation of their hearing. The study population is therefore a direct sample from the target population of adults presenting with subjective hearing loss to an outpatient ENT/audiology clinic and requesting evaluation of their hearing. Potential subjects must meet specific criteria during screening at the ENT clinic of the UZA before inclusion in the study. During the study, the subject has the right to drop out of the study at any time. A subject can be included in the study if the following criteria are met:

- All subjects must be 18–65 years old and must have signed an informed consent form.
- For inclusion in the normal hearing group, subjects must have a hearing threshold of ≤ 15 dB hearing level (HL) on pure tone average (PTA) at 500, 1000, 2000, and 4000 Hz, or ≤ 20 dB HL at one or more frequencies between 125 and 8000 Hz. The air–bone gap must be < 15 dB at 500, 1000, 2000, and 4000 Hz.
- For inclusion in the SNHL group, subjects must have a hearing threshold of > 15 dB HL on PTA at 500, 1000, 2000, and 4000 Hz, or > 20 dB HL at one or more frequencies between 125 and 8000 Hz. Patients exceeding 70 dB HL will be excluded.

If potential subjects present themselves with any cerebral condition (e.g., CVA), neurodegenerative diseases (e.g., multiple sclerosis, Parkinson’s disease), or insufficient language proficiency and/or cognition, they will be excluded from participation in the study. This will be verified based on the patient’s medical history. Patients with known middle ear pathology, Menière’s disease, or observed pathology during baseline examination will also be excluded.

If patients report alterations of their hearing at the time of the second visit, pure tone audiometry will be repeated and if there is deviation of ± 1 dB SD for these tests compared to the first visit, the patient will also be excluded.

### Test methods

#### ACC measurement

ACCs will be recorded using the procedure described by Vonck et al. [17]. The acoustic change stimuli consist of a reference tone with a base frequency of 1000, 2000, or 4000 Hz with a duration of 3000 ms gliding to a 300 ms target tone with a frequency that is 12% higher than the base frequency (i.e., 1000–1120 Hz; 2000–2240 Hz; 4000–4480 Hz). The same triplet of ACC stimuli will be used during the first and second visits. The three ACC stimuli will be presented at three base frequencies of 1000, 2000, and 4000 Hz. Sound stimuli will be presented monaurally, to the best hearing ear, through RadioEar DD45 supra-aural headphones at a level of 75 dB SPL in normal-hearing subjects or at maximum comfortable loudness (MCL) level in subjects with SNHL in order to attempt to correct for differences in loudness. This will result in stimulus presentation levels ranging from 75 dB SPL to MCL level with 100 dB SPL as the upper limit for those with SNHL.

Participants will be seated in a comfortable reclining chair in an electrically shielded, sound-attenuated booth and are allowed to watch a silent, captioned movie. Electrophysiological responses will be recorded by electrodes placed according to the 10–20 system using the Synergy Nicolet EDX evoked potential system. The active electrode will be placed at the vertex of the skull (Cz), the contralateral mastoid (A1/A2) will be used as the reference electrode and the ground electrode will be placed on the forehead. Eye movements and blinks will be monitored using electrodes above and below the eye, contralateral of the stimulated ear. Blink artefact rejection will be applied during the recordings. Responses will be recorded using a sampling frequency of 50 kHz and filtered from 0.01 to 100 Hz. For each recording, 100 accepted sweeps will be averaged to obtain individual grand averages. The total duration of three ACC recordings required for the prediction model is around 30 min. Only the best hearing ear will be assessed. For waveform analysis, the N1 latency, N1 amplitude, P2 latency, P2 amplitude, and N1-P2 amplitude of the ACC response will be determined.

#### Pure-tone audiometry

Pure-tone audiometry with air conduction is performed at 125, 250, 500, 1000, 2000, 3000, 4000, 6000, and 8000 Hz using a two-channel Interacoustics AC-40 audiometer and headphones. Bone conduction thresholds are tested at 250, 500, 1000, 2000, 3000, and 4000 Hz. Procedures and requirements for pure-tone air conduction and bone conduction threshold audiometry are according to ISO 8253–1:2010.

#### Speech reception in noise testing (SRT)

Speech in noise scores (in dB SNR) will be obtained according to two clinically validated procedures; the Leuven Intelligibility Sentences Test (LIST) [18] and the Nederlandse Vereniging voor Audiologie (NVA) Consonant–Vowel-Consonant (CVC) words [19].

#### Phoneme discrimination test (A§E discrimination test)

The Auditory Speech Sounds Evaluation (A§E®) is a software package containing a phoneme discrimination test used to determine the ability to discriminate two different phonemes [20]. This auditory test will solely be used as a secondary outcome measurement.

#### Questionnaire

All participants will be questioned about their hand dominance, language proficiency, and musical experience. Participants will be asked if they practice music and if so, how many hours they play per week and for how many years. In accordance with Vonck et al. (2021) and Van Heteren et al. (2022), a ‘musical experience score’ will be calculated by multiplying the average amount of musical experience in hours per week by the years of active engagement. A score of > 15 reflects significant musical engagement [17, 21].

### Analysis

#### Primary outcome measurements

The level of agreement between the predicted SRT by the original ACC prediction model and the actual SRT as determined using the LIST and NVA list will be assessed. The maximum limit of acceptable difference is set at ± 2 dB.

The level of agreement between the SRT calculated from the first ACC measurement (1st visit) and the SRT calculated from the second ACC measurement (2nd visit) will be assessed with Bland–Altman plots. The maximum limit of acceptable difference is set at ± 2 dB (2 × 1 dB SD).

#### Secondary outcome measurements

First, the intraclass correlation coefficient (ICC) and Pearson *r* will be determined between the ACC-predicted SRT and the measured SRT. Secondly, the correlation between the ACC N1 peak latencies and amplitudes and the A§E discrimination test will be determined. Lastly, the ICC and Pearson *r* between the SRT calculated by the first ACC prediction model (1st visit) and the SRT calculated by the second ACC prediction model (2nd visit) will be assessed.

### Sample size calculation

Based on the prediction model: SRT =  − 6.4 + 0.071*HL + 0.083*(ACC_latency_ – 100), with ACC_latency_ being the average of ACC recordings at three different base frequencies and HL being the PTA (average hearing loss for 1000, 2000, and 4000 Hz), we will have 6 variables in the model with SRT and HL in dB and ACC_latency_ in ms [14]. With a large effect size of *f*^2^ = 0.6667, an α = 0.01 and β = 0.99, we would need a total sample size of *n* = 62 (GPower 3.1, *F*-test linear multiple regression, *R*^2^ deviation from zero). This number is in line with the rule of thumb for sample size calculations for regression analyses of *n* = 10 per variable. We expect a potential patient dropout of 20% due to study visit duration. Therefore, a total of 78 subjects will be recruited.

The categories of severity of SNHL will be used as a blocking factor to establish an equal distribution of patients with various degrees of SNHL. The following 5 categories of SNHL degree, according to the international standards established by the World Health Organization, will be included: normal, mild, moderate, moderate-severe, and severe [22].

## Discussion

Each year, the UZA ENT department has over 10,000 hearing-related consultations. Based on this large number of patient visits, we expect the risk of low enrollment to be minimal. If study enrollment is lower than expected, the research group will reach out to the advisory board partners, which include patient organizations and hearing clinics. Study participants will be asked to visit twice for a study session of approximately 2 h for the first session and 1 h for the second. Since patients do not directly benefit from participation in this study, we expect a dropout of 20%. If the dropout is higher, the following fallback strategies will be considered. First, the duration of the first visit can be reduced by omitting the A§E phoneme discrimination test. This will reduce the duration of the study visit by approximately 10–15 min. Secondly, a subject can be excluded from the test–retest validity study from the study protocol. This will reduce the time investment of the participant by half, as the second visit is not required or mandatory anymore. With an effect size of 0.6667, an α = 0.01, and β = 0.80, we would need at least a remaining sample size of *n* = 30 (GPower 3.1, *t*-test means, difference between two dependent means (matched pairs)). If we expect a drop-out of 20% during the second visit, we need a total sample size of *n* = 36 in order to evaluate the test–retest reliability.

So far, attempts have been made to find a good model to predict speech in noise with different brainstem and cortical response paradigms, like the cortical auditory evoked potential (CAEP) and P300. However, due to a lack of clinically useful and consistent correlations, none of these objective measures have made it beyond research applications [23–27]. The prognostic ACC model is the first objective measure to predict speech in noise perception with high accuracy [14]. Since this prediction model was based on a study population of 37 adult subjects with only 13 subjects with SNHL, it is essential to validate this prediction model in an independent and large study population.

This project is highly innovative because the ACC prediction model can fill a notorious gap in the evaluation of hearing impairment. ENT departments and audiology clinics worldwide struggle with patients who have insufficient language proficiency or cognitive abilities to fulfill the conventional speech perception tests, children who are difficult to test, or cases where malingering is suspected. If this project proves that the ACC model provides a reliable prediction of speech in noise perception, it will provide the opportunity to perform adequate hearing evaluations in the aforementioned populations in whom no reliable audiometric assessments can be performed. The ACC model can improve hearing evaluation in the general population and patients will directly benefit from this diagnostic advancement, as it can provide access to better hearing rehabilitation.

## Data Availability

A scientific article will be written on the primary (and including secondary) outcomes of the study, according to the TRIPOD guidelines [28, 29], and results will be disseminated regardless of the magnitude or direction of the effect. The present study is not industry-initiated; therefore, there are no publication restrictions imposed by sponsors. Data collected within the study is disseminated to the public through publications and lectures. The raw collected pseudonymized data will be stored locally after closing the study but will not be made publicly available due to privacy regulations.

## Abbreviations

ACC: Acoustic Change Complex
A§E: Auditory Speech Sounds Evaluation
CAEP: Cortical auditory evoked potential
CI: Cochlear implant
CVC: Consonant-vowel-consonant
dB: Decibel
ENT: Ear Nose Throat
FDT: Frequency discrimination threshold
HL: Hearing level
Hz: Hertz
ICC: Intraclass correlation coefficient
LIST: Leuven Intelligibility Sentences Test
MCL: Maximum comfortable loudness
NVA: Nederlandse Vereniging voor Audiologie
PTA: Pure-tone average
SD: Standard deviation
SNHL: Sensorineural hearing loss
SNR: Signal-to-noise ratio
SPL: Sound pressure level
SRT: Speech reception threshold
UMC: University Medical Center
UZA: Antwerp University Hospital
